# Predictors of the rate of cognitive decline in older adults using machine learning

**DOI:** 10.1371/journal.pone.0280029

**Published:** 2023-03-03

**Authors:** Maryam Ahmadzadeh, Theodore David Cosco, John R. Best, Gregory James Christie, Steve DiPaola

**Affiliations:** 1 School of Interactive Arts and Technology, Simon Fraser University, Surrey, BC, Canada; 2 Gerontology Research Center, Simon Fraser University, Vancouver, BC, Canada; 3 Oxford Institute of Population Ageing, University of Oxford, Oxford, United Kingdom; University of Kurdistan Hewler, IRAQ

## Abstract

**Background:**

The longitudinal rates of cognitive decline among aging populations are heterogeneous. Few studies have investigated the possibility of implementing prognostic models to predict cognitive changes with the combination of categorical and continuous data from multiple domains.

**Objective:**

Implement a multivariate robust model to predict longitudinal cognitive changes over 12 years among older adults and to identify the most significant predictors of cognitive changes using machine learning techniques.

**Method:**

In total, data of 2733 participants aged 50–85 years from the English Longitudinal Study of Ageing are included. Two categories of cognitive changes were determined including minor cognitive decliners (2361 participants, 86.4%) and major cognitive decliners (372 participants, 13.6%) over 12 years from wave 2 (2004–2005) to wave 8 (2016–2017). Machine learning methods were used to implement the predictive models and to identify the predictors of cognitive decline using 43 baseline features from seven domains including sociodemographic, social engagement, health, physical functioning, psychological, health-related behaviors, and baseline cognitive tests.

**Results:**

The model predicted future major cognitive decliners from those with the minor cognitive decline with a relatively high performance. The overall AUC, sensitivity, and specificity of prediction were 72.84%, 78.23%, and 67.41%, respectively. Furthermore, the top 7 ranked features with an important role in predicting major vs minor cognitive decliners included age, employment status, socioeconomic status, self-rated memory changes, immediate word recall, the feeling of loneliness, and vigorous physical activity. In contrast, the five least important baseline features consisted of smoking, instrumental activities of daily living, eye disease, life satisfaction, and cardiovascular disease.

**Conclusion:**

The present study indicated the possibility of identifying individuals at high risk of future major cognitive decline as well as potential risk/protective factors of cognitive decline among older adults. The findings could assist in improving the effective interventions to delay cognitive decline among aging populations.

## Introduction

The longitudinal rates of cognitive decline among aging populations are heterogeneous [1, 2]. Stable to moderate decline in cognitive functioning is considered as the age-related cognitive decline which is a normal process of aging. In contrast, some individuals may exceed the expected cognitive decline with dementia at the extreme end [3, 4]. In the early stages of cognitive impairment, it is challenging to differentiate it from normal age-related cognitive decline due to the insidious onset of symptoms and similar early signs [5, 6]. In this regard, predicting longitudinal cognitive decline among older adults to discriminate future major cognitive decliners from those with stable/minor cognitive decline using only their baseline information is a research imperative. This could assist in targeting individuals who would benefit the most from the early interventions. Furthermore, the ability to identify the predictors of longitudinal cognitive decline in a broad range of aging could provide a great opportunity to delay/prevent cognitive decline among older adults [7].

Invasive biomarkers such as neuroimaging, genetic, and cerebrospinal fluid (CSF) sampling indicate promising findings for the early detection of cognitive impairments [8, 9]. However, the high cost of implementation and invasive nature of these modalities limit their widespread utilization [10]. By contrast, an ideal prognostic tool for the prediction of cognitive impairment is required to be practical in primary care settings. Such a tool needs to use non-invasive, easy-to-collect variables that are usually collected in community health care institutions [11, 12].

A large body of literature has examined various risk and protective factors of future cognitive functions such as health status, lifestyle factors, quality of life, and psychological contributors [13–15]. However, there are inconsistencies in the findings of the studies investigating the association between these factors and cognitive performance [16]. As an example, while evidence shows that factors such as education may have a cross-sectional impact on the level of cognitive function, this factor is not necessarily associated with the rate of cognitive change [17, 18].

The lack of consistency in the findings of the studies may be due to the fact that most existing research has investigated the effects of a single factor or a small number of potential factors. However, longitudinal cognitive change is a multidimensional and complicated process that is affected by a wide range of factors [19]. While there are mutual intercorrelations among potential factors, the correlations involving a limited number of potential factors might be affected by associations with other unmeasured variables. As such, including a large number of prognostic factors at the same time would reduce these confounding effects [19]. Furthermore, most previous studies have used statistical methods to study cognitive function [20, 21]. However, statistical methods are limited in their hypothesis and do not provide an assessment of the relative rankings of the potential predictors [2]. The potential predictors could be meaningful and valuable if appropriate data analysis techniques such as machine learning (ML) algorithms are used to evaluate their complex interactions. Unlike conventional statistical methods, ML algorithms can evaluate a large number of variables in big datasets, learning the combinations to predict the outcomes with high reliability [11, 22].

Accordingly, the objective of this study was to develop a robust multivariate predictive model. This machine learning-based model is used to predict the longitudinal rate of cognitive decline over 12 years of follow-up. This aimed to differentiate future major cognitive decliners from those with minor cognitive decline using their baseline information that is commonly collected in primary care settings. Following that, we aimed to identify and rank the most significant prognostic factors of longitudinal cognitive decline among healthy aging populations.

In the present study, episodic memory was used to estimate the changes in cognitive function in a population-based sample over 12 years. The decline in episodic memory is one of the most common and early signs of neurodegenerative changes in normal aging [23, 24]. Additionally, it is well documented that episodic memory changes are associated with various factors such as lifestyle, health status, and education that result in maintenance/decline of brain functioning [13, 24].

In our study, 43 potential predictors of cognitive trajectories were carefully selected from multiple domains, based on the findings of previous studies, systematic reviews, and meta-analysis papers as well as the knowledge of experts in this field [16, 25]. These potential predictors include 1) sociodemographic (i.e. age, gender, education, socioeconomic status), 2) social engagement (i.e. community activities, membership in social organizations), 3) health (i.e. cardiovascular (CVD) disease, non-CVD disease, general health, eyesight, hearing), 4) physical functioning (i.e. limitations with activities of daily living (ADL), mobility aid), 5) psychological (i.e. depressive symptoms, loneliness feeling), 6) health-related behaviors (i.e. physical activity, smoking, alcohol consumption), and 7) baseline cognitive factors (i.e. processing speed, self-rated memory, verbal fluency). These factors were carefully selected from the literature as the potential factors that contribute to cognitive aging trajectories [16, 25]. The predictive model was applied to the English Longitudinal Study of Ageing (ELSA) dataset that has covered multiple aspects of cognition along with the longitudinal measurements of cognitive function during broad bands of aging (50–85 years) [1]. The selection and tuning of the appropriate ML technique to build a predictive model with these types of data require in-depth knowledge and consideration. However, few, if any, studies have used advanced data mining techniques to analyze these types of psychosocial data with a lot of categorical and binary values [16, 21]. The result of this study could assist in promoting healthy aging in societies by implementing new policies and educating individuals.

## Methods

### Study population

The English Longitudinal Study of Ageing (ELSA) is an observational multidisciplinary panel study of older adults living in the England community [26]. In ELSA, data of participants are measured every 2 years by computer-assisted personal interviews (CAPI) and self-completion questionnaires. At the time of this study, ELSA includes eight waves over a follow-up period of up to 14 years from wave 1 (2002–2003) to wave 8 (2016–2017) [26].

In the present study, data of 2733 participants were selected spanning 12 years across wave 2 (2004–2005) to wave 8 (2016–2017). The exposure variables were derived from a broad range of domains including sociodemographic, social engagement, health, physical functioning, health behaviors, and cognitive tests at baseline (wave 2). Participants aged 50–85 years without dementia at the baseline assessment were included. The ethical approval of ELSA was obtained from the National Research Ethics Committee and all participants signed the informed consent (http://www.hra.nhs.uk/about-the-hra/our-committees/res/).

### Outcome variable

In the present study, verbal episodic memory was used to measure cognitive functioning in seven waves (wave 2- wave 8). According to the literature, verbal episodic memory is an early and common sign of age-related cognitive decline that is associated with the daily activities of aging populations [27, 28]. Immediate and delayed word recall tests (range 0–10) were used to assess episodic memory, where participants listened to 10 common words. Then, they were asked to recall as many words as possible, in any order immediately and after a short delay (about 5 minutes). The total episodic memory score was obtained using the correct number of recalled words in both tests (range 0–20). As such, a better episodic memory is indicated by a higher score. The word lists used in the present study were originally provided in the Health Retirement Study (HRS) [29], which presented good construct validity and consistency [30].

The participant-specific slopes of episodic memory from wave 2 (baseline) to wave 8 over 12 years of follow-up were estimated using the linear model with random intercepts and slopes [1]. This resulted in defining two groups of major and minor cognitive decliners. Participants with minor cognitive decline were those with predicted slopes more than 1 SD below the mean, while those with predicted slopes less than 1 SD below the mean were categorized as major cognitive decliners.

### Independent variables

A wide range of factors was derived both directly and indirectly from the collected data in ELSA. The factors were selected and categorized carefully based on their features and the literature [2, 14, 21, 31]. In total, the factors were categorized into 7 groups. For more information S1 Table.

#### 1. Sociodemographic

Sociodemographic factors included sex, ethnicity, education, age, marital status, employment status, occupation, and socioeconomic status. The variables of sex (male/female), and ethnicity (white/non-white) were binary with two groups. Based on the highest level of education, the variable of education was classified into five groups (no qualification, level 1 National Vocational Qualification (NVQ1), NVQ2, NVQ3, higher education but below degree, and NVQ 4/5). We categorized the variable of age into four groups (50–60, 60–70, 70–80, and 80–85 years) and marital status into six groups (married, remarried, single, legally separated, divorced, and widowed). Employment status was categorized into six groups (retired, employed, self-employed, unemployed, permanently sick or disabled, and looking after home or family). Based on the level of responsibility and intellectual requirement occupation was categorized into 8 groups (from never worked to higher managerial, and professional occupations). Finally, the collected economic variables were used to derive socioeconomic status. This measure was divided into deciles for the analysis [32].

#### 2. Social engagement

Social engagement included community activities, and social membership. Social membership that measured the participant’s membership to the question ‘‘How is organizations, societies, and clubs is classified into eight groups (i.e., resident groups, church or other religious groups, charitable associations, education, arts or music groups, or evening classes). Participants were asked to indicate the memberships that apply to them. The total number of selected membership options was considered as the final score of social membership for each individual (ranged 0 to 8). The variable of community activities measured the participation in different activities in the past 12 months. This measure was classified into 6 groups (i.e., engaging in a hobby or pastime, going on a holiday in the UK, holiday abroad, or outing). The final score of this measure was the sum of selected options (ranged 0–6).

#### 3. Health

Health factors included cardiovascular (CVD) disease, non-CVD disease, general health, eye disease, eyesight, and hearing. The variable of cardiovascular disease measured self-reported doctor-diagnosed heart condition that was divided into 9 groups (i.e., high blood pressure or hypertension, Angina, high cholesterol). Self-reported doctor-diagnosed chronic diseases were defined as non-CVD diseases and were classified into 7 groups (i.e., Parkinson’s disease, cancer or a malignant tumor, Asthma). The variable of general health indicated the participant’s self-response to the question ‘‘How is your health in general?”. Their response was divided into five-point scales (ranging from ‘‘poor” to ‘‘excellent”). Self-reported hearing measured the participant’s level of hearing and similar to the general health it was divided into five-point scales (ranging from ‘‘poor” to ‘‘excellent”). Self-rated eyesight was assessed by asking participants the three types of questions “Is your eyesight (using glasses or corrective lenses; if you use them) excellent/very good/good/fair/ or poor”, “How good is your eyesight for seeing things at a distance, like recognizing a friend across the street”, and “How good is your eyesight for seeing things up close, like reading ordinary newspaper print’. Their Responses were divided into five-point scales (ranging from ‘‘poor” to ‘‘excellent”). Finally, self-reported doctor-diagnosed eye condition was defined by the variable of eyesight which was divided into 4 groups (i.e., Glaucoma or suspected glaucoma, diabetic eye disease, macular degeneration)

#### 4. Physical functioning

Physical functioning consisted of limitations with activities of daily living (ADL), instrumental ADL, and mobility aid). According to the participant’s responses to physical difficulties of doing any of 10 basic activities of daily living (i.e., kneeling, climbing, lifting over 10 lbs., walking 100 yards) and 13 instrumental ADLs (i.e., walking across a room, taking medications, managing money, bathing, or showering) disabilities of the participants were measured. Those who reported difficulty with any of the activities were asked whether they have used any 7 types of walking aids (i.e., cane or walking stick, Zimmer frame or walker, manual wheelchair, electric wheelchair).

#### 5. Psychological

Psychological factors included depressive symptoms, loneliness, and life satisfaction. Depressive symptoms were measured using the 8-item Centre for Epidemiologic Studies Depression Scale (CES-D) that is validated to be used among elderlies in previous studies (ranged 0 to 8) [33]. The short form of the Revised University of California Los Angeles (UCLA) loneliness scale was used to assess the variable of loneliness [34]. This measure included 3-items. Finally, the Satisfaction with Life Scale (SWLS) included five items about overall life satisfaction that was developed in previous studies [35]. The response to this scale was divided into 7-point scales (ranging from strongly agree to strongly disagree).

#### 6. Health-related behaviors

Health-related behaviors included physical activity, smoking, and alcohol consumption. Physical activity was measured using self‐reported participation in three types of activities including vigorous, moderate, and low impact. More description of these three categories is provided in a previous study [36]. Self‐reported smoking status measured whether the participants smoke cigarettes at all nowadays. Their responses were either “yes” or “no”. The alcohol consumption factor captured the frequency that participants had an alcoholic drink in the last 12 months. The response to this scale was classified into 8-point scales (ranging from “Almost every day” to “not at all”.

#### 7. Cognitive tests

Baseline cognitive tests included health literacy, Self-rated memory change, self-rated memory, processing speed, verbal fluency, orientation, and word recall. Questions from the Mini-Mental State Examination were used to assess the orientation to the day, week, month, and year [37]. Immediate and delayed word recall tests were used based on the verbal memory learning task in the Health and Retirement Study [38]. In both tests, 10 words were presented to the participants, and they were asked to recall the words immediately or approximately after 5 minutes. The verbal fluency test is a common measurement in neuropsychological assessment where participants were asked to name as many animals as possible in 60 seconds. This measure assesses “how readily participants are able to think of words from a particular category” [39]. Processing speed was measured using a letter cancellation task from the National Study of Health and Development [40]. Participants were asked to “cross out as many of the 65 target letters (P and W) as possible in one minute”. The final score of processing speed captured the “total number of letters searched in one minute. A comprehension test that was previously developed in the International Adult Literacy Survey. was used to assess Health literacy [41]. This measure captured the required skill to understand and use health materials correctly. The variable of self-rated memory change indicated the participant’s self-response to the question ‘‘How is your perception of memory compared to 2 years ago?”. Their response was divided into three-point scales (better now, about the same, and worse now than it was then). Finally, self-rated memory measured participants’ self-response to their level of memory, and their response was divided into five-point scales (ranging from ‘‘poor” to ‘‘excellent”).

### Machine learning framework

Development of the prediction model for discrimination of future minor cognitive decliners from those with major cognitive decline involved multiple steps. First, the original ELSA dataset was randomly split into the train and test sets, where 85% and 15% of all data were allocated to the training and test set, respectively. In total, there were 2733 samples. As such, 2323 and 410 samples were used as the training and test data. Second, we applied several data pre-processing techniques including the normalization of data to have an average of 0 and a standard deviation of 1, encoding categorical data using the One-hot encoding method. Third, feature selection methods were applied to select the optimal subset of important features. Lastly, four different prediction models were individually implemented. In the following sections, a more in-depth description of the process is provided.

### Imbalanced data resampling

A problem with the classification of imbalanced data is that the number of samples in the minority class is too few, which results in decreasing the effectiveness of model learning. In the present study, the included dataset used to predict longitudinal cognitive changes was imbalanced where the prevalence of individuals with major cognitive changes was about 13.6%. Accordingly, we combined Synthetic Minority Oversampling Technique (SMOTE) with random under-sampling to resolve the imbalanced data problem in the training dataset before fitting a model.

Synthetic Minority Oversampling Technique (SMOTE) is an oversampling method that generates new synthetic samples from the minority class between the minority samples and their selected nearest neighbors [42]. This process is a type of data augmentation for tabular data that results in having larger and less specific decision regions. However, a disadvantage of the over-sampling method is that the majority class is not considered in generating the synthetic samples. This may cause creating an ambiguous sample if there is a strong overlap between the majority and minority classes.

In order to address the issue, we combined the SMOTE with under-sampling that has shown better performance compared to plain under-sampling [42]. Therefore, first, we oversampled the minority class, following that under-sampled the majority class to transform and balance the dataset. Furthermore, at the level of algorithm, the built-in parameters of each ML algorithm were tuned to deal with imbalanced data.

### Feature selection

Feature selection has a key role in machine learning-based models that removes irrelevant and redundant information from the original features. Appropriate feature selection would result in better interpretability, simplified modeling, shorter learning time, and enhanced generalization [43, 44]. In this regard, we used Ridge regression (RR) as a feature selection method to select the important features and rank each individual feature by reducing the size of the coefficients instead of setting them equal to zero. In the Ridge regression model, the L2 regularization technique is used that penalizes the L2-norm of the coefficients in linear regression. More specifically, the magnitude of feature coefficients is penalized while the error between actual and predicted values is minimized.

### ML-based models

In this study, multiple types of machine learning methods are provided. The * indicates the a including extreme gradient boosting (XGBoost), adaptive Boosting (AdaBoost), support vector machine (SVM), and multi-layer perceptron (MLP) neural network. These algorithms are selected due to their high performance in previous studies to implement prediction models for binary classification [45–48]. Further details of the optimal parameters of the selected machine learning algorithms are provided in S2 Table. In each individual algorithm of AdaBoost, XGBoost, SVM, and MLP the grid search technique was used separately to determine the optimal parameters based on the specific goals of this study. The grid search technique is a hyperparameter optimization method based on a defined subset of hyper-parameter space. The lower bound, upper bound, and the number of steps are required to specify the hyper-parameters in this technique [49]. All models were implemented with Python 3.7.6.

#### A. SVM

Support vector machine or SVM is a common approach for analyzing nonlinear data with small size and high dimensionality [50]. The basic theoretical foundation of this algorithm is to find an optimal partition hyperplane. This optimal hyperplane separates data into classes in a way that the blank area on both sides of the hyperplane is maximized [51]. The selection of this optimal hyperplane maximizes the ability of SVM to predict the classification of unseen data with high accuracy [52].

#### B. XGBoost

Extreme gradient boosting or XGBoost is an efficient and scalable supervised-learning algorithm used for regression and classification predictive modeling problems. XGBoost is an implementation of gradient-boosted decision trees designed for speed and performance [53]. Boosting algorithms are capable to convert weak learners (very simple models with slightly higher performance compared to random guessing) into strong learners [51]. In gradient boosting new models are created that predict the errors of prior models and then added together to make the final prediction. The reason behind calling it gradient boosting is due to the gradient descent algorithm minimizing the loss function when adding new models. However, gradient-boosting machines are generally slow in implementation because of sequential model training. Hence, they are not very scalable. Thus, XGBoost is focused on computational speed and reliable model performance in many classification tasks [46, 54]. XGBoost provides parallelization of tree construction using all CPU cores during training and distributed computing for training very large models using a cluster of machines. Furthermore, a regularization term is added to control the complexity of the tree, limit the growth of the tree, and the decline of the loss function. While the basic idea of XGBoost is to sum multiple tree classifiers, this algorithm avoids overfitting to some extent. XGBoost could be used for different types of tasks including classification, regression, and even feature ranking by using sequentially-built shallow decision trees to provide accurate results and a highly-scalable training method that avoids overfitting [47].

#### C. AdaBoost

Adaptive boosting or AdaBoost is a popular technique for generating ensembles that was developed by Freund and Schapire [55]. The main idea of AdaBoost is to implement a strong prediction rule by the combination of multiple relatively weak and inaccurate learners. These learners are combined through a weighted vote. In general, a weak learner has slightly higher performance compared to random guessing [56]. In AdaBoost, the weak learners are decision trees with a single split that are called decision stumps. Once the first decision stump is created, all observations are weighted the same. Then, in order to correct the previous error, higher weights were assigned to the observations that were classified incorrectly. Thereby, each subsequent weak learner was forced to concentrate on the incorrectly classified samples by the previous ones in the sequence. AdaBoost algorithms could be used for both classification and regression problems. Two important hyperparameters of ADAboost include the number of estimators and the learning rate. The number of estimators controlled the number of weak learners and learning rate controlled the contribution of the weak learners in the final combination.

#### D. MLP

**Fig 1:**
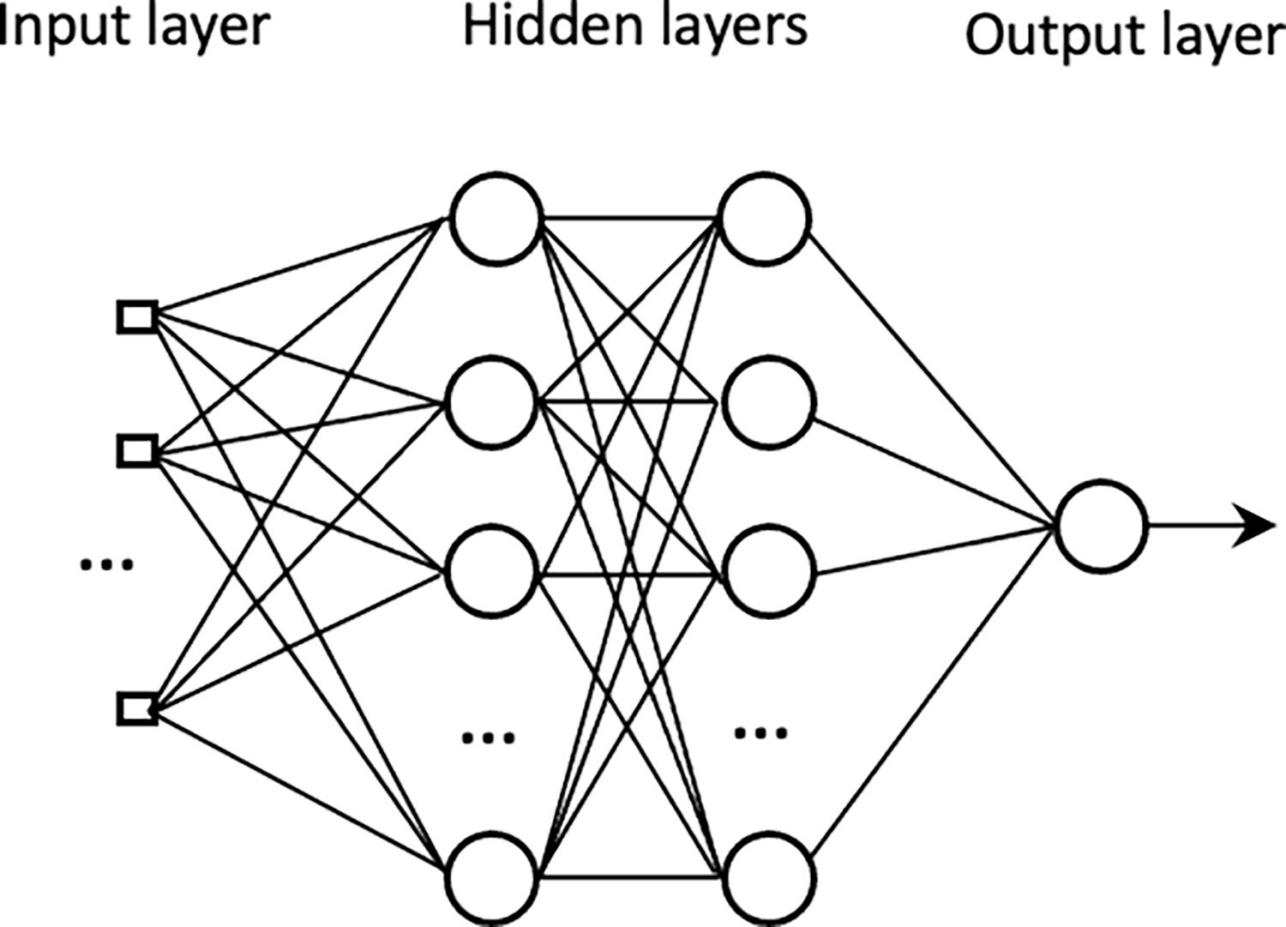
Structure of an MLP model with two hidden layers, and one output.

**Fig 2:**
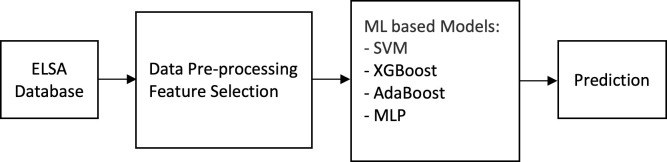
Framework of the proposed machine learning prediction model.

Multi-layer perceptron or MLP is a feed-forward neural network that consists of several layers of neurons to flow the information from the input layer to the output layer [57]. The connections between interacting neurons are weighted. The degree of correlation between the activity levels of the connected neuron is measured by weights. Back-propagation algorithms are commonly used to correct the weights from output layers backward and to train the neural network [45]. The performance MLP model is related to multiple parameters such as the numbers of hidden layers, neurons, learning rate, momentum, and the number of iterations. The speed and effectiveness of the MLP model are balanced by the learning rate and the momentum. In Fig 1, the architecture of an MLP neural network is represented. In this study, a four fully connected (FC) layers of the perceptron neural network was used. The sigmoid activation function was used for the node output (output layer of the model) where it takes a real-valued input and squashes it to range between 0 and 1. In contrast, the ReLU (Rectified Linear Unit) was used for the hidden layers, where it outputs the input directly if the output is positive, otherwise, it outputs the input zero. Besides, the “AdaBound” and “binary cross-entropy” were used as the optimizer and loss (cost) function to train the model. The grid search which is based on a defined subset of hyper-parameter space was used as the model hyperparameter optimization technique. In Fig 2, the framework of the proposed machine learning prediction model is presented.

### Evaluation metrics

After random distribution of the original ELSA dataset into the training and test sets the validation approach was applied to the training set. We used the repeated stratified 5-fold cross-validation method to train and validate the prediction models and to achieve a reliable measure. In repeated stratified 5-fold cross-validation, the original sample is randomly partitioned into 5 equal-sized subsets in a way that maintains the same class distribution in each subset. This could preserve the imbalanced class distribution in each fold and enforce the class distribution in each split of the data to match the distribution in the complete training dataset. Then, the validation process is repeated 3 times, with each of the 5 subsamples used exactly once as the validation data [21]. In this study, the goal was to ensure that each fold had the same proportion of major cognitive decline observations.

Three indexes of sensitivity (SEN), specificity (SPE), and area under the curve (AUC) are used to calculate the results of models for binary classification. The “sensitivity” indicates the ability of the model to determine major cognitive decliners, correctly. This measure is defined as *TP*/(*TP*+*FN*), where *TP* and *FN* stand for the proportion of true positive (i.e., minor cognitive decliners who are classified correctly) and false negative cases (i.e., major cognitive decliners who are classified as minor cognitive decliners), respectively. In contrast, “specificity” indicates the ability of the model to determine minor cognitive decliners, correctly. This measure is defined as *TN*/(*TN*+*FP*), where *TN* and *FP* stand for the proportion of true negative (i.e., minor cognitive decliners who are correctly classified) and false positive case (i.e., minor cognitive decliners who are classified as major cognitive decliners), respectively. The derived measure of AUC determines the inherent ability of the test to discriminate between individuals with minor and major cognitive decline. Another interpretation of AUC is “the average value of sensitivity for all the possible values of specificity” [58]. A higher score on these indexes indicates a better model performance. We did not include the index of accuracy as a performance metric. This measure does not provide meaningful information regarding the performance of classification models due to the unequal number of participants in two groups of minor and major cognitive decliners [59].

## Results

### Cognitive groups (major vs minor cognitive decliners)

**Fig 3:**
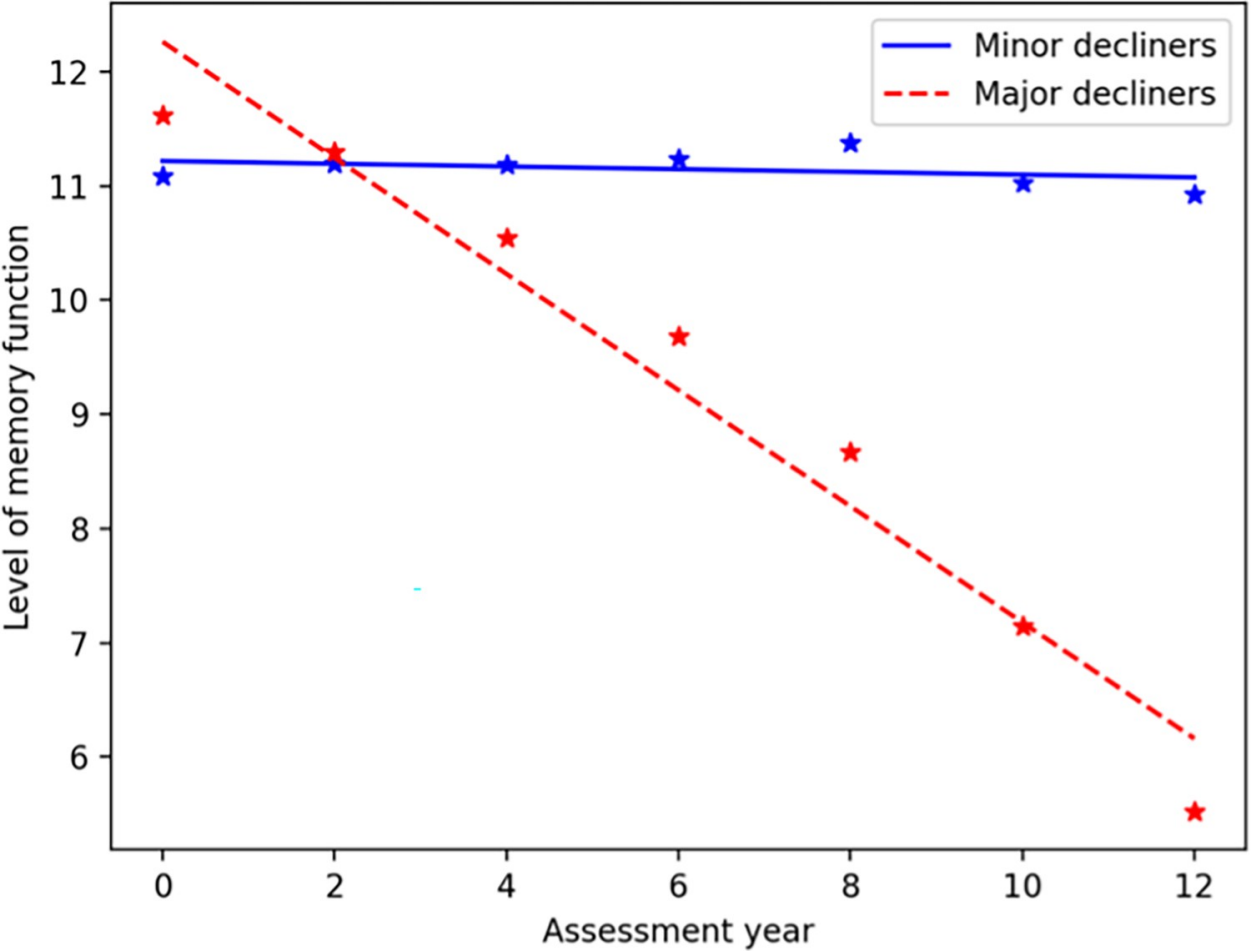
Two cognitive groups of major cognitive decliners (slope > 1 SD below mean) and minor cognitive decliners (slope ≤ 1 SD below mean) were determined based on the changes in the level of memory function (combination of immediate and delayed word recall tests) from baseline (year 1) to year 12. The * indicates the average memory function for participants in each group during the assessment year. The lines are fitted using the linear mixed model.

In total, 2733 participants are included in the present study where 2361 individuals (86.4%) were categorized as minor cognitive decliners and 372 individuals (13.6%) as major cognitive decliners. Fig 3 illustrated the estimated two cognitive groups with major cognitive decline (slope > 1 SD below mean) and minor cognitive decline (slope ≤ 1 SD below mean) based on the changes in the level of memory function. Memory function was measured using the combination of immediate and delayed word recall tests from baseline (year 1) to year 12. The slope (and intercept) of the estimated linear model for 12-year memory changes among minor and major cognitive decliner groups were -0.03 (intercept, 11.35) and -1.02 (intercept, 13.49) respectively.

### Participant characteristics

Descriptive results for characteristics of participants at baseline year are presented in Table 1. As shown, the characteristics of both minor and major cognitive decliner groups in seven domains including sociodemographic, social engagement, health, psychology, physical functioning, health-related behaviors, and baseline cognitive tests are presented. In the sociodemographic domain, compared to minor decliners, major decliners were more likely to be older, female, unmarried/divorced, relatively lower levels of education and employment, as well as lower levels of occupation status. However, participants in both groups were likely to have similar socioeconomic statuses. In the social engagement domain, those with participation in three or more community activities (i.e., engaging in a hobby or pastime, going on a holiday) were more likely to be major cognitive decliners. In contrast, the percentage of individuals who had the membership in four or more social organizations (i.e., resident groups, religious groups, charitable associations) were relatively similar in both cognitive groups. In the health domain, participants in the minor-decline group self-reported their health, eyesight, and hearing as good, very good, or excellent (vs fair or poor) compared to the major decliners. Furthermore, those with more than one CVD type disease (i.e., high cholesterol, hypertension, Angina) and eyesight were more likely to be major cognitive decliners. By contrast, the percentage of participants with one or more than one eye non-CVD type disease (i.e., Asthma, Cancer, or a malignant tumor) was relatively similar in both groups. In the physical functioning domain, major decliners were more likely to have at least one limitation with ADL (i.e., kneeling, climbing) and one limitation with IADL (i.e., showering, taking medications,). Furthermore, those in the group of major decline were more likely to need at least one type of mobility aid (i.e., cane or walking stick, manual wheelchair). In the psychology domain, those with relatively lower levels of depression symptoms (CES-D based depression), and lower level of loneliness feeling (UCLA based loneliness) was more likely to be in the group of minor cognitive decliners. However, in both groups, the level of life satisfaction (SWLS based life satisfaction) was relatively similar. In the health-related behaviors domain, participants in the minor cognitive decline group self-reported taking part in mild and moderate physical activities once or more per week. However, participants in the major were more likely to take part in vigorous physical activities once or more per week. Furthermore, the number of participants who drank more than one alcoholic drink per week during the last 12 months and current smokers were relatively higher in the group with minor cognitive decline. In the baseline cognitive tests domain, participants in the minor cognitive decline group self-reported their health literacy and eyesight as good, very good, or excellent (vs fair or poor) compared to the major decliners, their score of delayed word recall was likely higher as well. However, the scores on other cognitive tests including immediate word recall, time orientation, verbal fluency, self-rated memory change, processing speed, and health literacy were relatively similar among the participants of both cognitive groups.

**Table 1:** Characteristics of the participants in two groups of individuals with major and minor cognitive decline at baseline (wave 2 of ELSA dataset).

Domains and characteristics	Minor decliners (n = 2361)	Major decliners (n = 372)
Participants, %	86.39	13.61
**Sociodemographic**		
Age, [60–85] years, %	55.921	60.21
Gender, female, %	55.48	60.48
Marital status, married, %	72.97	65.86
Ethnicity, white, %	99	99.7
Education, NVQ4/5/Below degree, %	33.93	29.57
Employment status, Employed/Self-employed, %	43.07	26.07
Occupation, Professional/Intermediate occupations, %	42.18	37.63
Socioeconomic status (decile), mean (SD)	6.51 (2.69)	6.13 (2.71)
**Social engagement**		
Community activity, with ≥ 3 activities, %	54.34	62.36
Social membership, with ≥ 4 memberships, %	98.30	97.84
**Health**		
CVD, with ≥ 1 morbidity, %	33.41	38.17
Non-CVD, with ≥ 1 morbidity, %	13.30	13.44
Eye disease, with ≥ 1 morbidity, %	8.89	14.51
General health, good to excellent, %	85.90	83.06
Self-reported eyesight, good to excellent, %	70.35	66.66
Hearing, good to excellent, %	84.20	78.76
**Physical functioning**		
ADL, with ≥ 1 impairment, %	49.22	56.99
IADL, with ≥ 1 impairment, %	18.72	23.39
Mobility aid, with ≥ 1 type of mobility aids, %	4.45	9.41
**Psychology**		
CES-D based depression, mean (SD)	1.26 (1.76)	1.5 (1.97)
UCLA based loneliness, mean (SD)	3.93 (1.35)	4.08 (1.43)
SWLS based life satisfaction, mean (SD)	13.44 (5.83)	13.74 (6.14)
**Health-related behaviors**		
Mild physical activity, once or more than once/week, %	92.63	91.93
Moderate physical activity, once or more than once/week, %	85.09	81.45
Vigorous physical activity, once or more than once/week, %	73.91	79.30
Smoking, currently smoker, %	11.94	9.68
Alcohol consumption >1 alcoholic drink/week, %	66.83	66.39
**Baseline cognitive tests**		
Self-rated memory, good to excellent, %	70.26	69.35
Health literacy, mean (SD)	4.29 (0.59)	4.29 (0.61)
Processing speed, mean (SD)	19.87 (5.47)	19.31 (4.81)
Self-rated memory change, mean (SD)	2.30 (0.49)	2.29 (0.48)
Verbal fluency, mean (SD)	21.98 (6.06)	21.48 (6.13)
Time orientation, mean (SD)	1.15 (0.39)	1.18 (0.38)
Immediate word recall, mean (SD)	4.81 (1.53)	4.43 (1.46)
Delayed word recall, mean (SD)	6.00 (1.76)	5.73 (1.73)

CVD = Cardiovascular disease; ADL = Activities of daily living; IADL = Instrumental ADL; CES-D = Center for Epidemiologic Studies–Depression Scale score; UCLA = University of California Los Angeles; SWLS = Satisfaction with Life Scale; SD = Standard Deviation. CES-D based depression range 0–8, higher = more depression symptoms; UCLA based loneliness range 3–9, higher = more loneliness feelings; SWLS based life satisfaction range 5–35, higher: less satisfaction; Health literacy range 4–8, higher = worse health literacy; Processing speed range 0–64, reverse-coded: higher = better processing speed; Self-rated memory change range 1–3, higher = worse memory change; Verbal fluency range 0–63, reverse-coded: higher = better verbal fluency, Time orientation range 0–4, higher = worse time orientation, Immediate/delayed word recall range 0–10 reverse-coded: higher = better world recall.

### Performance of ML-based models

A total of 2733 samples was split to have 410 samples (15%) in the test set and 2323 samples (85%) in the training set. Following that, in the training stage, the pre-processing procedure including normalization and imbalanced data techniques are performed. Then, feature selection was applied using the Ridge regression method that resulted in the selection of optimal features. Following that, the performance of four ML-based predictive models in terms of three evaluation metrics including AUC, specificity, and sensitivity on the test data were obtained. The results are illustrated in Table 2. The best result was achieved using XGBoost with 72.84% AUC, 78.23% sensitivity, and 67.41% specificity overall. We further investigated the effects of the feature selection technique on the highest performance model (XGBoost). In this regard, the results of the XGBoost model in two conditions of using the original features (without any feature selection) and using the optimal subset of important features (with feature selection) were compared. The findings indicated that the performance of the model remained relatively stable. This shows the robustness of the XGBoost model and its less sensitivity to irrelevant and redundant features.

**Table 2:** Classification performance of four machine learning algorithms on the test set.

Models	Sensitivity (%)	Specificity (%)	AUC (%)
SVM	64.61	66.08	65.35
MLP	54.5	64.69	58.12
AdaBoost	66.15	61.86	63.51
XGBoost	78.23	67.41	72.84

AUC: Area under the curve.

### Top-ranking predictors of longitudinal cognitive changes

XGBoost is a tree-based ensemble learning algorithm that could be used for both classification and feature ranking [60]. Among all models, XGBoost showed the highest performance for discrimination of future major and minor cognitive decliners using the test set (see Table 2). As the next step, we used this algorithm as the feature ranking method to sort features according to the feature importance degree using Gini importance (mean decrease in impurity). In XGBoost, three measures of feature importance scores including ‘weight’, ‘gain’, and ‘cover’ could be used that represent the relative importance.

**Fig 4:**
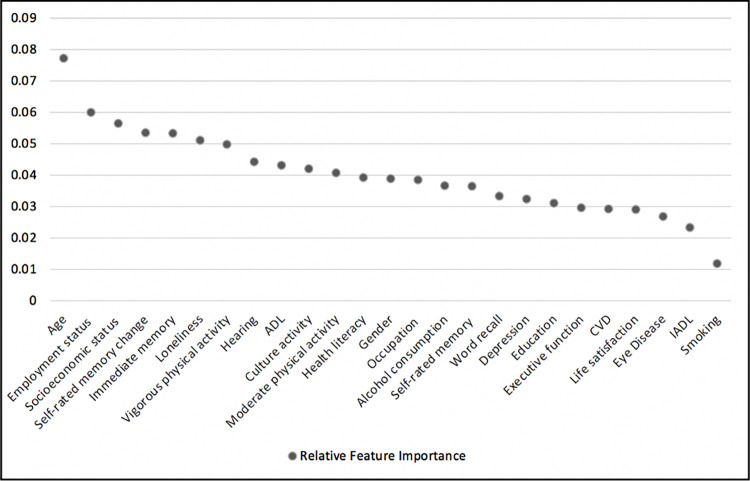
Top 25 ranked features with their score when predicting major cognitive decliners vs minor decliners using fitted XGBoost.

The ‘weight’ is a valid and commonly used measure to estimate the importance score of the features. This measure indicates how many times a feature is used to split the data across all trees. As such, in the present study, we used this measure for feature ranking [61]. Fig 4 illustrated the results of this method that determined the most influencing predictors on the longitudinal changes of memory function. Based on the results among all 43 baseline features the top 7 ranked features that had an important role in predicting major cognitive decliners vs minor decliners included age, employment status, socioeconomic status, self-rated memory changes, immediate word recall, feeling of loneliness, and vigorous physical activity. In contrast, the five least important baseline features consisted of smoking, instrumental activities of daily living, eye disease, life satisfaction, and cardiovascular disease.

## Discussion

In the real world, people are exposed to several risk and protective factors at the same time. While the impact of some individual factors on cognitive changes could be significant, the impacts of many other predictors are individually small. However, the multivariate accumulation of those small effects could be accounted for sizable influences [19]. Few studies have investigated the possibility of implementing prognostic models to predict future cognitive changes with the combination of categorical and continuous data from multiple domains such as sociodemographic, health, psychology, and cognition, simultaneously [16, 21].

In the current study, two related goals were investigated: 1) comparing advanced ML techniques for the prediction of longitudinal cognitive changes over 12 years of follow-up among older adults using advanced ML techniques, 2) identification of the most important predictors of longitudinal cognitive changes. In this regard, we determined two categories of longitudinal cognitive changes among adults aged 50–85 years including those with minor and major cognitive decline. While the majority of the total sample (86.4%) showed a minor cognitive decline, the minority of individuals (13.6%) showed a more rapid and major decline in their cognition.

Following that, we implemented our predictive model to discriminate future minor cognitive decliners from those with major cognitive decline over 12 years of follow-up with their baseline information. We initially derived 43 baseline features from seven domains (i.e., sociodemographic, social engagement, health, physical functioning, psychological, health-related behaviors, and baseline cognitive tests). These baseline features were carefully selected based on the findings of previous studies, systematic reviews, and meta-analysis papers as well as the knowledge of experts in this field [16, 25]. According to the findings, our model predicted future major versus minor cognitive decliners with the overall AUC, sensitivity, and specificity of 72.84%, 78.23%, and 67.41%, respectively. These findings indicate the possibility of using our predictive model to determine individuals at high risk for major cognitive decline in the future.

We also aimed to identify the most important predictors of future cognitive changes. XGBoost was used as an efficient feature ranking method to score and sort features based on their degree of importance. XGBoost as a feature ranking method has been used in the field of bioinformatics with high performance [53, 62, 63]. The top 7 ranked features that appeared to be protective of rapid cognitive decline included lower age, higher employment status, higher socioeconomic status, better self-rated memory changes compared to the past, higher performance in immediate word recall test, less feeling of loneliness, and more engagement in vigorous physical activities. On the other side, the five least important baseline features consisted of smoking, instrumental activities of daily living, eye disease, life satisfaction, and cardiovascular disease. While few studies have investigated the possibility of implementing prognostic models using several domains, the included feature in our study have been reviewed individually and separately in previous studies. For example, according to the literature, there is an association between the lower level of educational attainment and the incidence of cognitive decline and dementia [36, 38]. However, we did not find any predictive effect of educational attainment on cognitive decline. Consistent with our findings, previous studies found a relationship between socioeconomic status and cognitive functioning as well as the risk of dementia [64, 65]. A recent systematic review and meta-analysis of longitudinal studies indicated the positive association between loneliness and increased risk of dementia [66]. Similarly, the findings of another review showed the negative impact of loneliness on cognitive function [67]. The present study adds to this by indicating that less feeling of loneliness could be protective against rapid cognitive decline among healthy adults. Furthermore, the findings of a systematic review suggested that exercise is a promising nonpharmaceutical factor that could prevent age-related cognitive decline [68]. Our study indicated that vigorous physical activities (i.e., running, swimming, cycling, aerobics, or gym workout) could also be protective against rapid cognitive decline.

Regarding the technical considerations of our approach, feature selection is a regularization technique that is applied to data to decrease noisy features, maintain the significant features, and remove redundant features This process results in making the model faster, more interpretable, and prevents overfitting [44]. To further investigate the effects of the feature selection on the results of our model, we applied the XGBoost model to the training set with the initial features (without any feature selection). After tuning the parameters of XGBoost, we investigated the performance of the model on the test set. The results indicated that the performance remained relatively stable. This shows the robustness of the XGBoost model and its less sensitivity to irrelevant and redundant features [46].

In the present study, there are several limitations. First, self-report questionnaires were used to assess factors such as social engagements, physical activities, and health status which could have been affected by recall bias. Second, participants have relatively higher levels of socioeconomic status, occupation, and educational attainment compared to the normal community-based populations. This may result in decreasing the generalizability of our findings. Third, while cognitive functioning is a multifaceted construct, changes in the verbal episodic memory were considered as the output cognitive changes. However, according to the literature episodic memory has been a key factor in cognitive aging [24, 69]. Future studies are recommended to assess other cognitive domains such as visual or semantic memory, processing speed, and executive function.

Finally, this study was focused on using sociodemographic, social, health, physical functioning, cognitive, and psychological variables. However, literature shows that other types of brain modalities such as biological, clinical, neuroimaging, and genetic data could provide valuable information about the changes in the functionality and structure of the brain across time. It is recommended that in future studies the integration of these modalities be considered [20, 21]. In contrast, one strength of our study is the long-term follow-up across a total time frame of 12 years. This long period of time allows us to measure the gradual changes in cognitive function that happen through time. Besides, our investigated population-based cohort included a large sample of participants belonging to both groups with mild/stable cognitive changes and those with more rapid cognitive decline. In comparison with experimental and clinical data, the results of our study were more generalizable due to the population-based nature of the cohorts.

In the present study, we showed using our predictive model to identify those at high risk for major cognitive decline in the future. Furthermore, the identified predictors could improve the counseling of elderlies and caregivers and develop the planning of cognitive care and effective interventions to delay cognitive decline among aging populations.

## Supporting information

S1 Table Supporting information about the selected factors from each of the seven domains (including sociodemographic, social engagement, health, psychology, physical functioning, health-related behaviors, and baseline cognitive tests). (DOCX)

S2 Table The hyper-parameter setting of different ML algorithms. (DOCX)
