# Supplementary material for: Predictors of the rate of cognitive decline in older adults using machine learning
Source: PLoS One. 2023 Mar 3;18(3):e0280029. doi: 10.1371/journal.pone.0280029 (PMC9983884; doi:10.1371/journal.pone.0280029)
Supplement: S1 Table — (DOCX) [file pone.0280029.s001.docx]

**S1 Table. Supporting Information about the selected factors from each of the seven domains (including sociodemographic, social engagement, health, psychology, physical functioning, health-related behaviors, and baseline cognitive tests)**

| **1. Sociodemographic** | | | | | |
| --- | --- | --- | --- | --- | --- |
| **Variable** | **Label of variable** | **Value of variable** | **Sub-variables** | **Value of subvariables** | **Explanation** |
| Age | Respondent's current age | Value = 1  Label = [50-60) Value = 2  Label = [60-70) Value = 3  Label = [70-80) Value = 4  Label = [80-85] | _ | Integer:  [50-85] | Their age is classified into 4 groups. |
| Sex | Respondent's gender | Value = 1  Label = Male Value = 2  Label = Female | _ | _ | _ |
| Marital status | Respondent's current legal marital status | Value = 1.0  Label = Married Value = 2.0  Label=Remarried Value = 3.0  Label = Single Value = 4.0  Label = Legally separated Value = 5.0  Label = Divorced Value = 6.0  Label = Widowed | _ | _ | _ |
| Ethnicity | Respondent's ethnicity | Value = 1  Label = White Value = 2  Label = Non-white | _ | _ | _ |
| Education |  | Value = 1.0  Label=NVQ4/NV5 Value = 2.0  Label = below degree Value = 3.0  Label = NVQ3  Value = 4.0  Label = NVQ2  Value = 5.0  Label = NVQ1  Value = 6.0  Label = Foreign Value = 7.0  Label = None | _ | _ | _ |
| Employment status | The best description of the current situation | Value = 1.0  Label = Retired Value = 2.0  Label = Employed Value = 3.0  Label = Self-employed Value = 4.0  Label=Unemployed Value = 5.0  Label = Permanently sick  Value = 6.0  Label = Looking after home | _ | _ | _ |
| Occupation | Respondent's occupation. | Value = 1.0  Label = Higher managerial and professional occupations Value = 2.0  Label = professional occupations Value = 3.0  Label = Intermediate occupations Value = 4.0  Label = Small employers  Value = 5.0  Label = Technical occupations Value = 6.0  Label = Semi-routine occupations Value = 7.0  Label = Routine occupations Value = 8.0  Label = Never worked | _ | _ | _ |
| Socio-  economic status | Deciles of BU total (non-pension) wealth | Numeric (1, 2, ...,10) | _ | _ | _ |
| **2. Social Engagement** | | | | | |
| Community activities | Community activities during the last 12 months | 0,1,2,…,6 | Respondent has a hobby or pastime | Value = 0 Label = No Value = 1 Label = Yes | Sum of 6-items |
|  |  |  | taken a holiday in the UK in the last 12 months | ‘’ |  |
|  |  |  | taken a holiday abroad in the last 12 months | ‘’ |  |
|  |  |  | gone on a daytrip or outing in the last 12 months | ‘’ |  |
|  |  |  | uses the internet and/or email | ‘’ |  |
|  |  |  | owns a mobile phone | ‘’ |  |
| Social membership | Membership of social organization or club | 0,1,2,…,8 | member of a political party/ environmental groups | ‘’ | Sum of 8-items |
|  |  |  | member of resident groups, neighbourhood | ‘’ |  |
|  |  |  | member of a church or other religious group | ‘’ |  |
|  |  |  | member of charitable associations | ‘’ |  |
|  |  |  | member of education, arts, or music groups | ‘’ |  |
|  |  |  | member of social clubs | ‘’ |  |
|  |  |  | a member of sports clubs, gyms, exercise classes | ‘’ |  |
|  |  |  | member of other organisations or societies | ‘’ |  |
| **3. Health** | | | | | |
| CVD disease | Cardiovascular disease | 0,1,…,9 | Diagnosed cardiovascular condition newly reported at W2 (1st mention) | Value = 96.0  Label = None of these Value = 1.0  Label = High blood pressure Value = 2.0  Label = Angina Value = 3.0  Label = A heart attack  Value = 4.0  Label = Congestive heart failure Value = 5.0  Label = heart murmur Value = 6.0  Label = abnormal heart rhythm Value = 7.0  Label = Diabetes or high blood  Value = 8.0  Label = stroke  Value = 9.0  Label = High cholesterol | Sum of 9-item |
|  |  |  | (2nd mention) | ‘’ |  |
|  |  |  | (3rd mention) | ‘’ |  |
|  |  |  | (4th mention) | ‘’ |  |
|  |  |  | (5th mention) | ‘’ |  |
|  |  |  | (6th mention) | ‘’ |  |
|  |  |  | (7th mention) | ‘’ |  |
|  |  |  | (8th mention) | ‘’ |  |
|  |  |  | (9th mention) | ‘’ |  |
| non-CVD disease | Non-cardiovascular disease | 0,1,…,7 | Diagnosed chronic condition newly reported at W2 (1st mention) | Value = 96.0  Label = None of these Value = 1.0  Label = Chronic lung disease  Value = 2.0  Label = Asthma Value = 3.0  Label = Arthritis  Value = 4.0  Label = Osteoporosis  Value = 5.0  Label = Cancer  Value = 6.0  Label = Parkinson's disease Value = 7.0  Label = Any emotional, nervous or psychiatric problems | Sum of 4-item |
|  |  |  | (2nd mention) | ‘’ |  |
|  |  |  | (3rd mention) | ‘’ |  |
|  |  |  | (4th mention) | ‘’ |  |
| non-CVD &/or CVD disease | non-CVD and/or CVD disease | 0,1,2,3 | Diagnosed any types of CVD and/or non-CVD disease(s) reported at W2 | Value = 0.0  Label = no disease  Value = 1.0  Label = CVD disease Value = 2.0  Label = non-CVD disease Value = 3.0  Label = both disease types | _ |
| Eyesight | Self-reported eyesight | 0,1,2,3 | Self-reported eyesight (while using lenses, if appropriate) | Value = 1.0  Label = excellent Value = 2.0  Label = very good Value = 3.0  Label = good Value = 4.0  Label = fair Value = 5.0  Label = poor | Sum of associated 3-item |
|  |  |  | Eyesight for recognition of friend across street | ‘’ |  |
|  |  |  | Eyesight for reading ordinary newspaper print | ‘’ |  |
| Eye disease |  | 0,1,…,6 | Diagnosed eye condition reported at Wave 1 (1st mention) | Value = 96.0  Label = None of these Value = 1.0  Label = Glaucoma  Value = 2.0  Label = diabetic eye disease Value = 3.0  Label = macular degeneration Value = 4.0  Label = cataracts | Sum of associated 3-item |
|  |  |  | (2nd mention) | ‘’ |  |
|  |  |  | (3rd mention) | ‘’ |  |
| Hearing | Self-reported hearing | 1,…,5 | Self-reported hearing (while using hearing aid if appropriate) | ‘’ | _ |
| General health | Self-reported general health | 1,…,5 | Self-reported general health | ‘’ | _ |
| **4. Physical Functioning** | | | | | |
| IADL | Instrumental activities of daily living (IADL) | 0,1,2,…,13 | Activity has problem with due to health/physical problem (1st mention) | Value = 1.0  Label = Dressing Value = 2.0  Label = Walking  Value = 3.0  Label = Bathing  Value = 4.0  Label = Eating, Value = 5.0  Label = Getting in/ out of bed Value = 6.0  Label = Using the toilet  Value = 7.0  Label = Using a map  Value = 8.0  Label = Preparing a hot meal Value = 9.0  Label = Shopping  Value = 10.0  Label = Making telephone calls Value = 11.0  Label = Taking medications Value = 12.0  Label = Doing work around the house  Value = 13.0  Label = Managing money  Value = 96.0  Label = None | Sum of 13-items |
|  |  |  | (2nd mention) | ‘’ |  |
|  |  |  | (3rd mention) | ‘’ |  |
|  |  |  | (4th mention) | ‘’ |  |
|  |  |  | (5th mention) | ‘’ |  |
|  |  |  | (6th mention) | ‘’ |  |
|  |  |  | Activity has problem with due to health/physical problem (7th mention) | ‘’ |  |
|  |  |  | Activity has problem with due to health/physical problem (8th mention) | ‘’ |  |
|  |  |  | Activity has problem with due to health/physical problem (9th mention) | ‘’ |  |
|  |  |  | Activity has problem with due to health/physical problem (10th mention) | ‘’ |  |
|  |  |  | Activity has problem with due to health/physical problem (11th mention) | ‘’ |  |
|  |  |  | Activity has problem with due to health/physical problem (12th mention) | ‘’ |  |
|  |  |  | Activity has problem with due to health/physical problem (13th mention) | ‘’ |  |
| ADL | Activities of daily living (ADL) | 0,1,2,…,10 | Activity has problem with due to health/physical problem (1st mention) | Value = 96.0  Label = None Value = 1.0  Label = Walking 100 yards Value = 2.0  Label = Sitting for about two hours Value = 3.0  Label = Getting up from a chair  Value = 4.0  Label = Climbing several stairs without resting Value = 5.0  Label = Climbing one flight of stairs without resting Value = 6.0  Label = Stooping, kneeling, or crouching Value = 7.0  Label = Reaching or extending arms  Value = 8.0  Label = Pulling/pushing large objects  Value = 9.0  Label = Lifting/carrying over 10 lbs Value = 10.0  Label = Picking up a coin | Sum of 10-items |
|  |  |  | (2nd mention) | ‘’ |  |
|  |  |  | (3rd mention) | ‘’ |  |
|  |  |  | (4th mention) | ‘’ |  |
|  |  |  | (5th mention) | ‘’ |  |
|  |  |  | (6th mention) | ‘’ |  |
|  |  |  | (7th mention) | ‘’ |  |
|  |  |  | (8th mention) | ‘’ |  |
|  |  |  | (9th mention) | ‘’ |  |
|  |  |  | (10th mention) | ‘’ |  |
| Sum of IADL& ADL | _ | 0,1,2,…,23 | _ | _ | Sum of 2-items |
| Mobility aid | Mobility aid | 0,1,…,7 | Value = 96.0 Label = None of these Value = 1.0 Label = A cane or walking stick Value = 2.0 Label = A zimmer frame or walker Value = 3.0 Label = A manual wheelchair Value = 4.0 Label = An electric wheelchair Value = 5.0 Label = A buggy or scooter Value = 6.0 Label = Special eating utensils Value = 7.0 Label = A personal alarm Value = 8.0 Label = Elbow crutches Value = -9.0 Label = Refusal Value = -8.0 Label = Don't know Value = -1.0 Label = Not applicable | | Sum of 7-items |
|  |  |  | ‘’ | |  |
|  |  |  | ‘’ | |  |
|  |  |  | ‘’ | |  |
|  |  |  | ‘’ | |  |
|  |  |  | ‘’ | |  |
| **5. Psychology** | | | | | |
| CES-D based depression | Depression | 0,1,…,8 | Whether felt depressed much of the time during past week | Value = 1.0 Label = Yes Value = 2.0 Label = No | CES-D based depression |
|  |  |  | Whether felt everything they did during past week was an effort | Value = 1.0 Label = Yes Value = 2.0 Label = No |  |
|  |  |  | Whether felt their sleep was restless during past week | Value = 1.0 Label = Yes Value = 2.0 Label = No |  |
|  |  |  | Whether was happy much of the time during past week | Value = 1.0 Label = Yes Value = 2.0 Label = No |  |
|  |  |  | Whether felt lonely much of the time during past week | Value = 1.0 Label = Yes Value = 2.0 Label = No |  |
|  |  |  | Whether enjoyed life much of the time during past week | Value = 1.0 Label = Yes Value = 2.0 Label = No |  |
|  |  |  | Whether felt sad much of the time during past week | Value = 1.0 Label = Yes Value = 2.0 Label = No |  |
|  |  |  | Whether could not get going much of the time during past week | Value = 1.0 Label = Yes Value = 2.0 Label = No |  |
| UCLA based loneliness | Loneliness | 3,4,..,9 | How often feels they lack companionship | Value = 1.0 Label = Hardly ever or never Value = 2.0 Label = Some of the time Value = 3.0  Label = Often | UCLA based loneliness |
|  |  |  | How often feels left out | ‘’ |  |
| Satisfaction with Life Scale (SWLS) | Life satisfaction | 5,6,…,35 | Whether satisfied with their job | Value = 1.0  Label = Strongly agree Value = 2.0  Label = Agree Value = 3.0  Label = Disagree Value = 4.0  Label = Strongly disagree | The sum of associated 5 SWLS items  (reversed the negative items) |
|  |  |  | Whether feels their job is physically demanding | ‘’ |  |
|  |  |  | Whether receives the recognition they deserve in their work | ‘’ |  |
|  |  |  | Whether feels their salary is adequate | ‘’ |  |
|  |  |  | Whether feels their job promotion prospects are poor | ‘’ |  |
|  |  |  | Whether feels their job security is poor | ‘’ |  |
|  |  |  | Whether feels under constant pressure due to a heavy workload | ‘’ |  |
|  |  |  | Whether feels they have little freedom to decide how to do their work | ‘’ |  |
|  |  |  | Whether feels they have the opportunity to develop new skills | ‘’ |  |
|  |  |  | Whether feels they receive adequate support in difficult situations | ‘’ |  |
|  |  |  | Whether feels they have control over what happens in most situations | ‘’ |  |
|  |  |  | Whether feels they have to work fast | ‘’ |  |
| **6. Health-related behaviors** | | | | | |
| Smoking | Whether smokes cigarettes at all nowadays | Value = 1.0  Label = Yes Value = 2.0  Label = No | _ | _ | _ |
| Alcohol | How often respondent has had an alcoholic drink during the last 12 months | Value = 1.0  Label = Almost every day Value = 2.0  Label = 5-6 days a week Value = 3.0  Label = 3-4 days a week Value = 4.0  Label = Once or twice a week Value = 5.0  Label = Once or twice a month Value = 6.0  Label = Once every couple of months Value = 7.0  Label = Once or twice a year Value = 8.0  Label = Not at all in the last 12 months | _ | _ | _ |
| Vigorous physical activity | Frequency does vigorous sports or activities | Value = 1.0  Label = more than once a week Value = 2.0  Label = once a week Value = 3.0  Label 1-3 times a month, Value = 4.0  Label = hardly ever/never | _ | _ | _ |
| Moderate physical activity | Frequency does moderate sports or activities | ‘’ | _ | _ | _ |
| Mild physical activity | Frequency does mild sports or activities | ‘’ | _ | _ | _ |
| Physical activity (any intensity) | _ | _ | Frequency does vigorous sports or activities | Value = 1.0  Label = more than once a week Value = 2.0  Label = once a week Value = 3.0  Label = 1-3 times a month Value = 4.0  Label = hardly ever | Sum of 3-itmes |
|  |  |  | Frequency does moderate sports or activities | ‘’ |  |
|  |  |  | Frequency does mild sports or activities | ‘’ |  |
| **7. Cognitive test** | | | | | |
| Immediate word recall | Immediate word recall | 0,1,…,10 | Number of words recalled immediately | Integer: range (0-10) | _ |
| Delayed word recall | Delayed word recall | 0,1,…,10 | Number of words recalled after delay | Integer: range (0-10) | _ |
| Word recall | Word recall | 0,1,…,20 | Number of words recalled immediately | Integer: range (0-20) | _ |
| Time orientation: day of month | Time orientation: day of month | 1,2 | Whether correct day of month given | Value = 1.0  Label = Correct answer Value = 2.0  Label = Incorrect answer: | _ |
| Time orientation: month | Time orientation: month | 1,2 | Whether correct month given | ‘’ | _ |
| Time orientation: year | Time orientation: year | 1,2 | Whether correct year given | ‘’ | _ |
| Time orientation: day of week | Time orientation: day of week | 1,2 | Whether correct day given | ‘’ | _ |
| Time orientation: overall score | Time orientation: overall score | 0,1,2,3,4 | Computed: score from date questions | Integer: range (0-4) | _ |
| Verbal (semantic) fluency | Verbal (semantic) fluency | 0,10,..,63 | Number of animals mentioned | Integer | _ |
| Self-rated memory | Self-rated memory | 1,2,3,4,5 | Self-rated memory | Value = 1.0  Label = excellent Value = 2.0  Label = very good Value = 3.0  Label = good Value = 4.0  Label = fair Value = 5.0  Label = poor | _ |
| Self-rated memory change | Self-rated memory change | 1,2,3 | Perception of memory compared to 2 years ago | Value = 1.0  Label = better now Value = 2.0  Label = about the same, Value = 3.0  Label = or worse now | _ |
| Processing speed | Processing speed | 0,10,..,64 | Number of animals mentioned (fluency) | Integer | _ |
| Health literacy | Health literacy | 4,5,..,8 | Literacy test: Qu1: Whether correct number of days given | Value = 1.0 Label = Correct answer: seven Value = 2.0 Label = Incorrect answer: any other response | Sum of 4-items |
|  |  |  | Literacy test: Qu2: Whether three correct situations given | ‘’ | _ |
|  |  |  | Literacy test: Qu3: Whether one correct condition given for taking tablet | ‘’ | _ |
|  |  |  | Literacy test: Qu4: Whether one correct condition given for not taking tablet | ‘’ | _ |
