# Supplementary material for: Predictors of the rate of cognitive decline in older adults using machine learning
Source: PLoS One. 2023 Mar 3;18(3):e0280029. doi: 10.1371/journal.pone.0280029 (PMC9983884; doi:10.1371/journal.pone.0280029)
Supplement: S2 Table — (DOCX) [file pone.0280029.s002.docx]

**S2 Table.** The hyper-parameter setting of different ML algorithms

| **﻿Method** | **﻿ Hyper-parameters** |
| --- | --- |
| Adaptive Boosting (AdaBoost) | algorithm = 'SAMME.R'  learning_rate = 0.003  n_estimators = 250 |
| Extreme Gradient Boosting  (XGB) | learning_rate = 0.1  max_depth = 3  subsample = 0.5 colsample_bytree = 0.5  n_estimators = 100  gamma = 0  min_child_weight = 3 nthread = 4  seed = 10  objective = 'binary:logistic'  eval_metric ='aucpr' |
| SVM | kernel = 'linear'  degree = 6  gamma = 'scale'  class_weight = 'balanced' |
| Multi-layer perceptron (MLP) | ﻿loss = 'binary_crossentropy'  activation function of hidden layer = ‘relu’  activation function of output layer = ‘sigmoid’  optimizer = AdaBound  metrics = ‘CosineSimilarity  kernel_initializer = ‘glorot_uniform’  batch_size = 512 epochs = 1000 |
